# Influencing factors of prognosis in HIV infected individuals undergoing surgical treatment for colorectal cancer

**DOI:** 10.3389/fonc.2026.1749639

**Published:** 2026-03-23

**Authors:** Xia Li, Chao Zhang, Guanzhen Wang, Minhong Mou, Mengmeng Zhu, Qianxi Guo, Yuxuan Yang, Ruyi Tian, Jianning Deng, Lei Huang

**Affiliations:** 1 School of Management, Shanxi Medical University, Taiyuan, China; 2 Senior Department of Infectious Diseases, Chinese PLA General Hospital, National Clinical Research Center for Infectious Diseases, Beijing, China; 3 Department of Pathology, The Fourth People’s Hospital of Nanning, Nanning, China; 4 Medical School of Chinese PLA, Beijing, China; 5 Department of Infectious Diseases, The Fourth People’s Hospital of Nanning, Nanning, China

**Keywords:** colorectal cancer, human immunodeficiency virus, nomogram model, prediction model, prognosis

## Abstract

**Objective:**

This study aimed to identify potential independent predictors of 1-year postoperative overall survival (OS) in people living with the Human Immunodeficiency Virus (HIV) who had undergone curative resection for colorectal cancer (CRC), and to construct a predictive model as an initial risk stratification tool.

**Methods:**

A total of 54 HIV-infected patients who underwent radical resection for CRC at the Fourth People’s Hospital of Nanning, Guangxi Zhuang Autonomous Region from January 2020 to December 2024 were enrolled. Their demographic data, pathological characteristics, and preoperative serological test indicators were collected. COX regression analyses were performed to identify potential independent factors affecting prognosis. A nomogram model predicting 1-year OS was subsequently constructed and internally validated.

**Results:**

BMI, interval time from HIV diagnosis to antiretroviral therapy (ART) initiation, CA19-9, CA125, and BASO% were potential independent prognostic factors for 1-year postoperative OS in HIV-infected patients with CRC. The nomogram constructed based on these variables exhibited an area under the curve (AUC) of 0.935, and the optimism value from bootstrap internal validation was 0.005.

**Conclusion:**

The nomogram derived from the identified potential independent factors demonstrated acceptable discriminative ability in internal validation and could serve as a visual preliminary risk stratification tool for 1-year prognosis in HIV-infected patients after curative CRC surgery.

## 1 Introduction

Acquired Immunodeficiency Syndrome (AIDS) is a chronic infectious disease caused by the Human Immunodeficiency Virus (HIV), which poses a serious threat to global health (1). Colorectal cancer (CRC) is also a major worldwide health problem (2). In China, by the end of 2024, there were estimated 749,839 people living with HIV, 605,178 AIDS patients, and 491,437 HIV-related deaths, with AIDS ranking as the leading cause of deaths among notifiable infectious diseases (3). In 2022 alone, CRC accounted for 517,000 new cases and 240,000 deaths in China, ranking second in incidence and fourth in mortality among malignant tumors (4).

HIV mainly targets CD4^+^ T lymphocytes, leading to progressive immune dysfunction and a higher risk of malignant tumors in HIV-infected individuals. However, with the widespread use of antiretroviral therapy (ART) (5–7), viral replication in the plasma of HIV-infected individuals can be effectively controlled, rendering it undetectable in the plasma (8). Consequently, the incidence of AIDS-defining cancers (ADCs) has decreased, and the life expectancy of patients has significantly increased. However, the incidence of non-AIDS-defining cancers, such as Hodgkin’s lymphoma, liver cancer, lung cancer, perianal tumors, and CRC, has relatively increased in HIV-infected individuals (9).

Compared with HIV-negative CRC patients, HIV-infected patients with CRC (HIV-CRC patients) are typically diagnosed at a younger age (10), present with more advanced tumor stages, greater invasiveness, faster progression, a higher likelihood of distant metastasis, and shorter overall survival (OS) (11–13). Due to HIV infection, HIV-CRC patients exhibit impaired intestinal epithelial barrier function, gut microbiota dysbiosis, and disrupted immune function (14). Even with ART treatment, the count of gastrointestinal CD4^+^ T lymphocytes rarely returns to normal levels (15). At present, the treatment strategies for HIV-CRC patients are mainly based on surgical resection, supplemented by radiotherapy, chemotherapy, targeted therapy, and immunotherapy, which are broadly similar to those for HIV-negative CRC patients (16, 17). However, studies have indicated that the risk of postoperative death in HIV-CRC patients is 3.12 times higher than that in HIV-negative individuals (12), and their OS is significantly reduced (11). Therefore, identifying potential prognostic risk factors and implementing targeted interventions are crucial for improving the survival rate of HIV-CRC patients after surgery. Nevertheless, there is not only a scarcity of research on the postoperative prognosis of HIV-CRC patients but also a lack of intuitive and practical clinical tools for predicting outcomes.

To address this gap, we conducted a comprehensive analysis that integrated demographic data, pathological characteristics, and preoperative serological parameters in HIV-CRC patients using a multimodal statistical approach. Concurrently, potential prognostic factors were identified and used to develop a nomogram-based clinical risk stratification tool, which aims to facilitate the early identification of HIV-CRC patients at high risk of a poor prognosis.

## 2 Patients and methods

### 2.1 Ethical approval of the study protocol

This study was a retrospective analysis of medical records. The research protocol was approved by the Ethics Review Committee of the Fifth Medical Center of the PLA General Hospital (KY-2025-3-40-1) and the Medical Ethics Committee of the Fourth People’s Hospital of Nanning (2024–96). The study adhered to the ethical principles outlined in the International Ethical Guidelines for Biomedical Research Involving Human Subjects by the Council for International Organizations of Medical Sciences and the Declaration of Helsinki by the World Medical Association. Due to the retrospective study design, both the Ethics Review Committee of the Fifth Medical Center of the PLA General Hospital (KY-2025-3-40-1) and the Medical Ethics Committee of the Fourth People’s Hospital of Nanning (2024-96) approved a waiver of written informed consent. All patient data were obtained from the hospital database for research purposes only.

### 2.2 Patients

**Figure 1:**
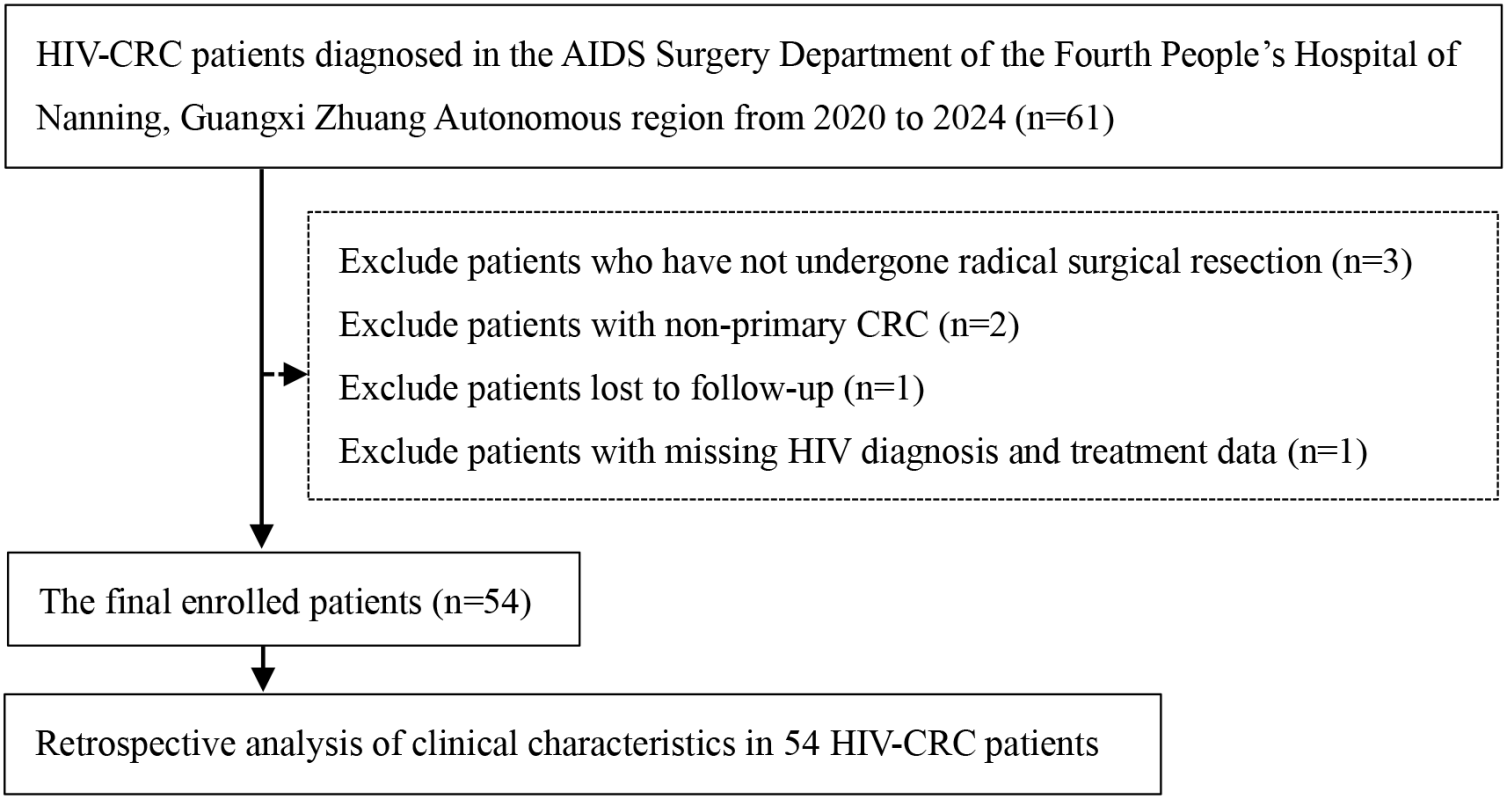
HIV-CRC patients selection flowchart.

HIV-infected patients diagnosed with primary CRC who underwent radical resection at the Fourth People’s Hospital of Nanning, Guangxi Zhuang Autonomous region from January 2020 to December 2024 were enrolled. The inclusion criteria were based on the “Chinese Guidelines for the Diagnosis and Treatment of AIDS (2024)” and the “Chinese Society of Clinical Oncology Guidelines for the Diagnosis and Treatment of Colorectal Cancer (2024)”. Inclusion criteria comprised: (1) age 18–75 years; (2) positive anti-HIV antibody status; and (3) diagnosed with CRC. A total of 61 HIV-CRC patients were enrolled. Exclusion criteria included: (1) absence of radical surgical resection; (2) non-primary CRC; (3) loss to follow-up; (4) missing data regarding HIV diagnosis and treatment. Finally, a total of 54 patients with HIV-CRC were enrolled. The screening process for HIV-CRC patients is shown as follows (**Figure 1**).

### 2.3 Data and grouping

The study endpoint was 1-year postoperative overall OS, defined as all-cause mortality within 1 year after surgery. Survival time was calculated as the number of months from the date of surgery to the date of death or the last follow-up via telephone (January 19, 2025). For patients with a follow-up duration of less than 1 year who remained alive at the last follow-up date, their last known survival status was used for analysis. Demographic data, pathological characteristics, and the most recent preoperative laboratory test results, including liver function, renal function, complete blood count, myocardial enzymes, and coagulation parameters, were collected for all patients. HIV-related information, including the date of HIV diagnosis, initial ART prescription record, and ART regimen, was obtained from patient self-reports and outpatient pharmacy records. The interval time from HIV diagnosis to ART initiation was defined as the number of months from the date of HIV confirmation to the date of the first ART prescription. The interval time from ART initiation to surgery was defined as the number of months from the date of the first ART prescription to the date of surgery. To describe baseline characteristics, patients were divided into the survival group (n=39) and the non-survival group (n=15) according to their 1-year postoperative survival status. The survival group included 8 patients with less than 1 year of follow-up who were alive at the last follow-up; these 8 patients were treated as censored data in the subsequent survival analysis.

### 2.4 Statistical analysis

Statistical analysis and graphical representation were performed using SPSS 27.0.1 and R 4.5.0 software. Categorical variables were expressed as [n (%)] using the chi-square test. An independent samples t-test was used to analyze continuous variables with normal distribution and homogeneity of variance, which were reported as ( $\bar{x}\pm s$). The Mann-Whitney U test was used to analyze the continuous variables that were either non-normally distributed or exhibited heterogeneity of variance, which were presented as [*M* (*Q_L_*, *Q_U_*)]. Survival curves were constructed using the Kaplan−Meier method, and between-group comparisons were performed using the Log−rank test. The Cox proportional hazards regression model was applied to calculate Hazard Ratios (HR) and 95% Confidence Intervals (CI). Variables with *P* < 0.05 in univariate analysis were included in the multivariate analysis, which was conducted using the backward stepwise method based on the likelihood ratio test. Internal validation was performed using the bootstrap method. The R 4.5.0 software was used to develop and visualize the nomogram prediction model, plot Receiver Operating Characteristic (ROC) curves and calibration curves, and perform Decision Curve Analysis (DCA). The two−sided *P* < 0.05 was considered statistically significant.

## 3 Results

### 3.1 Baseline characteristics of HIV-CRC patients

Baseline characteristics of the enrolled HIV-CRC patients are summarized in **Tables 1**–**3** according to data type. Categorical variables are summarized in **Table 1**. Comparisons of baseline characteristics between HIV-CRC patients in the survival group and the non-survival group showed statistically significant differences in the following variables: preoperative ART history (*χ²* = 4.22, *P* = 0.040), preoperative anemia severity (*χ²* = 8.87, *P* = 0.031), tumor differentiation grade (*χ²* = 5.34, *P* = 0.021), postoperative chemotherapy (*χ²* = 5.78, *P* = 0.016), and distant metastasis (M stage) (*χ²* = 7.86, *P* = 0.005). Continuous variables with normal distribution and homogeneity of variance are presented in **Table 2**. Continuous variables that were either non-normally distributed or exhibited heterogeneity of variance are presented in **Table 3**. The survival group demonstrated a significantly higher median BMI (*Z* = 2.43, *P* = 0.015), shorter median time from HIV diagnosis to ART initiation (*Z* = 3.93, *P* < 0.001), lower median CA125 level (*Z* = 3.64, *P* < 0.001), higher median ALB level (*Z* = 2.68, *P* = 0.007), lower median α1-AT level (*Z* = 2.13, *P* = 0.033), higher median HGB level (*Z* = 2.15, *P* = 0.032), higher median BASO% (*Z* = 2.63, *P* = 0.008), and lower median hsCRP level (*Z* = 2.32, *P* = 0.020) compared with the non-survival group.

**Table 1:** Baseline characteristics of HIV-CRC patients.

Variable	Total (*n* = 54)	Survivor group (*n* = 39)	Non-survivor group (*n* = 15)	*χ^2^* values	*P* values
	[*n* (%)]				
Gender					
Female	14 (25.9)	9 (23.1)	5 (33.3)	0.18	0.672
Male	40 (74.1)	30 (76.9)	10 (66.7)		
Preoperative ART					
No	13 (24.1)	6 (15.4)	7 (46.7)	5.60	0.133
NRTI + INSTI	4 (7.4)	3 (7.7)	1 (6.7)		
NRTI + PI	7 (13.0)	6 (15.4)	1 (6.7)		
NRTI + NNRTI	30 (55.6)	24 (61.5)	6 (40.0)		
Preoperative ART					
No	13 (24.1)	6 (15.4)	7 (46.7)	4.22	0.040
Yes	41 (75.9)	33 (84.6)	8 (53.3)		
Hypertension					
No	42 (77.8)	30 (76.9)	12 (80)	0.06	0.808
Yes	12 (22.2)	9 (23.1)	3 (20)		
Diabetes mellitus					
No	50 (92.6)	36 (92.3)	14 (93.3)	0.02	0.897
Yes	4 (7.4)	3 (7.7)	1 (6.7)		
Smoking					
No	36 (66.7)	26 (66.7)	10 (66.7)	0.00	1.000
Yes	18 (33.3)	13 (33.3)	5 (33.3)		
Alcohol abuse					
No	43 (79.6)	32 (82.1)	11 (73.3)	0.11	0.737
Yes	11 (20.4)	7 (17.9)	4 (26.7)		
Intestinal obstruction					
No	36 (66.7)	28 (71.8)	8 (53.3)	1.66	0.197
Yes	18 (33.3)	11 (28.2)	7 (46.7)		
Abdominal pain					
No	26 (48.1)	19 (48.7)	7 (46.7)	0.02	0.893
Yes	28 (51.9)	20 (51.3)	8 (53.3)		
Hematochezia					
No	24 (44.4)	15 (38.5)	9 (60.0)	2.04	0.154
Yes	30 (55.6)	24 (61.5)	6 (40.0)		
Anemia					
No	9 (16.7)	8 (20.5)	1 (6.7)	8.87	0.031
Mild	19 (35.2)	17 (43.6)	2 (13.3)		
Moderate	17 (31.5)	9 (23.1)	8 (53.3)		
Severe	9 (16.7)	5 (12.8)	4 (26.7)		
Tumor location					
Left	27 (50.0)	19 (48.7)	8 (53.3)	0.40	0.819
Right	14 (25.9)	11 (28.2)	3 (20.0)		
Rectal	13 (24.1)	9 (23.1)	4 (26.7)		
Histological type					
Adenocarcinoma	1 (1.9)	0 (0)	1 (6.7)	0.25	0.617
Squamous cell carcinoma	53 (98.1)	39 (100)	14 (93.3)		
Degree of differentiation					
Low	7 (13.0)	2 (5.1)	5 (33.3)	5.34	0.021
Moderate	47 (87.0)	37 (94.9)	10 (66.7)		
CRM status					
No	52 (96.3)	38 (97.4)	14 (93.3)	0.46	0.498
Yes	2 (3.7)	1 (2.6)	1 (6.7)		
PNI					
No	49 (90.7)	34 (87.2)	15 (100)	0.87	0.351
Yes	5 (9.3)	5 (12.8)	0 (0)		
Chemotherapy					
No	29 (53.7)	17 (43.6)	12 (80.0)	5.78	0.016
Yes	25 (46.3)	22 (56.4)	3 (20.0)		
AJCC					
I-II	26 (48.1)	19 (48.7)	7 (46.7)	0.02	0.893
III-IV	28 (51.9)	20 (51.3)	8 (53.3)		
T stage					
1	3 (5.6)	2 (5.1)	1 (6.7)	4.82	0.185
2	11 (20.4)	9 (23.1)	2 (13.3)		
3	34 (63.0)	26 (66.7)	8 (53.3)		
4	6 (11.1)	2 (5.1)	4 (26.7)		
N stage					
0	30 (55.6)	20 (51.3)	10 (66.7)	1.07	0.585
1	15 (27.8)	12 (30.8)	3 (20.0)		
2	9 (16.7)	7 (17.9)	2 (13.3)		
M stage					
0	46 (85.2)	37 (94.9)	9 (60.0)	7.86	0.005
1	8 (14.8)	2 (5.1)	6 (40.0)		

NRTI, nucleoside reverse transcriptase inhibitor; INSTI, integrase strand transfer inhibitor; PI, Protease Inhibitor; NNRTI, non-nucleoside reverse transcriptase inhibitor; CRM, Circumferential resection margin; PNI, Perineural invasion; AJCC stage, American Joint Committee on Cancer stage; TNM stage, Tumor Node Metastasis stage.

**Table 2:** Baseline characteristics of HIV-CRC patients.

Variable	Total (*n* = 54)	Survivor group (*n* = 39)	Non-survivor group (*n* = 15)	*T* values	*P* values
	( $\bar{x}\pm s$)				
TP	66.42 ± 7.96	66.90 ± 7.43	65.17 ± 9.37	0.72	0.478
RBC	3.66 ± 0.77	3.68 ± 0.80	3.62 ± 0.71	0.24	0.814
FAR	1.47 ± 0.25	1.48 ± 0.24	1.46 ± 0.31	0.26	0.794

TP, Total Protein; RBC, Red Blood Cell; FAR, fibrinogen-to-albumin ratio.

**Table 3:** Baseline characteristics of HIV-CRC patients.

Variable	Total (*n* = 54)	Survivor group (*n* = 39)	Non-survivor group (*n* = 15)	*Z* values	*P* values
	[*M* (*Q_L_, Q_U_*)]				
Age	65.5 (52.75, 70)	64 (53, 68)	68 (49, 72)	0.70	0.480
BMI	20.91 (19.18, 22.6)	21.67 (19.72, 22.86)	19.71 (18.26, 20)	2.43	0.015
Interval time from HIV diagnosis to ART initiation (months)	0 (0, 0.25)	0 (0, 0)	1 (0, 24)	3.93	<0.001
Interval time from ART initiation to surgery (months)	34 (1, 96)	46 (3, 96)	4 (0, 78)	1.62	0.105
Interval time from surgery to discharge (months)	18 (13.75, 26.25)	17 (14, 28)	20 (10, 22)	0.57	0.568
Lymph node metastasis rate	0 (0, 0.11)	0 (0, 0.09)	0 (0, 0.17)	0.37	0.709
CD4^+^ T	385.5 (208.25, 468.2)	400 (224, 466)	327 (188, 542)	0.59	0.556
CD4^+^/CD8^+^ T	0.64 (0.49, 0.99)	0.67 (0.52, 1.01)	0.58 (0.37, 0.82)	1.11	0.267
CEA	4.02 (2.3, 10.14)	4.4 (2.35, 11.16)	2.87 (1.65, 7.5)	0.65	0.518
CA19-9	13.69 (7.68, 27.34)	11.66 (7.3, 19.14)	19.22 (11.91, 40.6)	1.56	0.117
CA125	16.51 (10.44, 34.04)	12.4 (7.69, 23.3)	37.37 (20.6, 93.62)	3.64	<0.001
LDH	180 (152.23, 226.6)	174.7 (150.8, 224.82)	186.9 (164.7, 239)	1.18	0.239
FERR	128 (16.18, 318.65)	116.9 (16.2, 309.1)	137.1 (14.6, 425.7)	0.27	0.787
ALP	76.05 (63.53, 105.5)	76.4 (62.7, 105)	75.7 (63.8, 140.1)	0.58	0.562
TBIL	6.65 (4.99, 8.48)	6.2 (4.8, 8.1)	7.6 (5, 9.3)	1.03	0.301
ALB	39.05 (34.78, 41.75)	39.8 (36.3, 43.3)	35.1 (31.2, 39.1)	2.68	0.007
α1-AT	156.05 (138.08, 189.43)	152.2 (136.8, 173.47)	191.6 (139.8, 206.7)	2.13	0.033
Fib	3.48 (2.76, 4.30)	3.48 (2.7, 3.9)	3.48 (2.98, 4.92)	1.35	0.176
WBC	5.35 (4.48, 6.75)	5.3 (4.2, 6.4)	5.8 (4.8, 9)	1.47	0.142
HGB	99 (83, 114.5)	106 (88, 118)	86 (83, 102)	2.15	0.032
PLT	268 (205.5, 319.5)	259 (215, 313)	277 (173, 361)	0.20	0.839
BASO%	0.4 (0.28, 0.6)	0.5 (0.3, 0.6)	0.3 (0.2, 0.4)	2.63	0.008
hsCRP	4.74 (1.48, 5.2)	3.6 (1.3, 5.2)	5.2 (5.2, 5.2)	2.32	0.020
CRP	5.8 (4.8, 19.41)	4.8 (4.8, 19.2)	9.7 (5.7, 32)	1.82	0.069
NLR	35.98 (30.29, 49.25)	35.83 (31.33, 47)	36.13 (28.67, 53)	0.28	0.779
LMR	0.07 (0.05, 0.1)	0.07 (0.05, 0.1)	0.05 (0.03, 0.08)	1.78	0.075
PLR	106.67 (81.4, 153.88)	107.94 (92.97, 153.33)	104.8 (69.33, 172.5)	0.51	0.609

CD4^+^T, CD4 positive T lymphocytes; CD4^+^/CD8^+^T, CD4 positive/CD8 positive T lymphocytes ratio; CEA, carcinoembryonic antigen; CA19-9, carbohydrate antigen 19-9; CA125, cancer antigen 125; LDH, lactate dehydrogenase; FERR, ferritin; ALP, alkaline phosphatase; TBIL, Total Bilirubin; ALB, albumin; α1-AT, α1-antitrypsin; Fib, fibrinogen; WBC, White Blood Cell; HGB, hemoglobin; PLT, Platelet; BASO%, basophil percentage; hsCRP, high-sensitivity C-reactive protein; NLR, neutrophil-to-lymphocyte ratio; LMR, lymphocyte-to-monocyte ratio; PLR, platelet-to-lymphocyte ratio.

### 3.2 Analysis of potential independent influencing factors affecting 1-year OS after surgery in HIV-CRC patients

Univariate Cox proportional hazards regression analysis was performed on data collected from HIV-CRC patients. The results showed that BMI, interval time from HIV diagnosis to ART initiation, preoperative ART history, tumor differentiation grade, postoperative chemotherapy, M stage, CA19-9, CA125, ALB, α1-AT, BASO%, and hsCRP were associated with 1-year OS after surgery (*P* < 0.05). These variables were subsequently included in multivariate Cox proportional hazards regression analysis. Variable selection was conducted using the backward stepwise method based on the likelihood ratio test, which is more robust for small sample sizes and tends to select slightly more complex models with better fitting performance. Multivariate analysis demonstrated that BMI (HR = 0.727, 95%*CI* = 0.569~0.930, *P* = 0.011), interval time from HIV diagnosis to ART initiation (HR = 1.063, 95%*CI* = 1.030~1.097, *P* < 0.001), CA19-9 (HR = 1.002, 95%*CI* = 1.000~1.004, *P* = 0.018), CA125 (HR = 1.004, 95%*CI* = 1.000~1.007, *P* = 0.048), and BASO% (HR = 0.005, 95%*CI* =<0.001~0.197, *P* = 0.005) were potential independent influencing factors for 1-year OS in HIV-CRC patients. Among them, BMI and BASO% were protective factors, while interval time from HIV diagnosis to ART initiation, CA19-9, and CA125 were risk factors (**Table 4**).

**Table 4:** Analysis of independent factors for 1-year OS after surgery in HIV-CRC patients.

Variable	Univariate analysis		Multivariate analysis	
	HR (95%*CI*)	*P* values	HR (95%*CI*)	*P* values
Gender				
Female	1			
Male	1.516 (0.517~4.444)	0.448		
Age (year)	1.001 (0.953~1.053)	0.954		
BMI	0.811 (0.659~0.999)	0.049	0.727 (0.569~0.930)	0.011
Interval time from HIV diagnosis to ART initiation (months)	1.040 (1.013~1.067)	0.003	1.063 (1.030~1.097)	<0.001
Preoperative ART				
No	1			
NRTI + INSTI	0.450 (0.055~3.663)	0.455		
NRTI + PI	0.231 (0.028~1.879)	0.171		
NRTI + NNRTI	0.351 (0.118~1.044)	0.060		
Preoperative ART				
No	1			
Yes	0.338 (0.122~0.933)	0.036		
Interval time from ART initiation to surgery (months)	0.994 (0.984~1.004)	0.257		
Hypertension				
No	1			
Yes	0.892 (0.252~3.162)	0.859		
Diabetes mellitus				
No	1			
Yes	0.860 (0.113~6.548)	0.884		
Smoking				
No	1			
Yes	1.006 (0.344~2.947)	0.991		
Alcohol abuse				
No	1			
Yes	1.655 (0.527~5.202)	0.389		
Intestinal obstruction				
No	1			
Yes	2.056 (0.744~5.681)	0.165		
Abdominal pain				
No	1			
Yes	1.142 (0.413~3.155)	0.798		
Hematochezia				
No	1			
Yes	0.442 (0.157~1.245)	0.122		
Anemia				
No	1			
Mild	0.974 (0.088~10.740)	0.983		
Moderate	4.701 (0.588~37.610)	0.145		
Severe	4.728 (0.528~42.377)	0.165		
Tumor location				
Left	1			
Right	0.662 (0.176~2.497)	0.543		
Rectal	0.918 (0.276~3.052)	0.890		
Histological type				
Squamous cell carcinoma	1			
Histological type	0.263 (0.034~2.028)	0.200		
Degree of differentiation				
Low	1			
Moderate	0.246 (0.083~0.729)	0.011		
CRM status				
No	1			
Yes	1.669 (0.219~12.718)	0.621		
PNI				
No	1			
Yes	0.042 (0.000~59.129)	0.391		
Chemotherapy				
No	1			
Yes	0.246 (0.069~0.872)	0.030		
AJCC stage				
I-II	1			
III-IV	1.058 (0.383~2.920)	0.914		
T stage				
1	1			
2	0.466 (0.042~5.148)	0.534		
3	0.623 (0.078~4.991)	0.656		
4	2.138 (0.237~19.266)	0.498		
N stage				
0	1			
1	0.565 (0.155~2.056)	0.386		
2	0.625 (0.137~2.858)	0.544		
M stage				
0	1			
1	6.032 (2.097~17.353)	<0.001		
Lymph node metastasis rate	1.709 (0.375~7.794)	0.489		
CD4^+^ T	0.999 (0.997~1.002)	0.700		
CD4^+^/CD8^+^ T	0.450 (0.130~1.558)	0.208		
CEA	1.004 (0.999~1.009)	0.117		
CA19-9	1.002 (1.000~1.004)	0.010	1.002 (1.000~1.004)	0.018
CA125	1.004 (1.001~1.007)	0.013	1.004 (1.000~1.007)	0.048
LDH	1.007 (1.000~1.015)	0.056		
FERR	1.002 (1.000~1.004)	0.062		
ALP	1.006 (1.000~1.012)	0.061		
TBIL	1.032 (0.898~1.186)	0.660		
TP	0.978 (0.917~1.042)	0.492		
ALB	0.919 (0.852~0.992)	0.029		
α1-AT	1.022 (1.007~1.038)	0.004		
Fib	1.507 (0.919~2.471)	0.104		
WBC	1.119 (0.983~1.274)	0.089		
RBC	0.881 (0.451~1.721)	0.710		
HGB	0.974 (0.946~1.002)	0.070		
PLT	1.001 (0.996~1.007)	0.609		
BASO%	0.019 (0.001~0.303)	0.005	0.005 (<0.001~0.197)	0.005
hsCRP	1.460 (1.029~2.072)	0.034		
CRP	1.015 (0.997~1.033)	0.103		
NLR	1.016 (0.990~1.042)	0.232		
LMR	0.000 (0.000~23.347)	0.101		
PLR	1.001 (0.995~1.008)	0.684		
FAR	0.705 (0.091~5.462)	0.738		

BMI, body mass index; NRTI, nucleoside reverse transcriptase inhibitor; INSTI, integrase strand transfer inhibitor; PI, Protease Inhibitor; NNRTI, non-nucleoside reverse transcriptase inhibitor; CRM, Circumferential resection margin; PNI, Perineural invasion; AJCC stage, American Joint Committee on Cancer stage; TNM stage, Tumor Node Metastasis stage; CD4^+^T, CD4 positive T lymphocytes; CD4^+^/CD8^+^T, CD4 positive/CD8 positive T lymphocytes ratio; CEA, carcinoembryonic antigen; CA19-9, carbohydrate antigen 19-9; CA125, cancer antigen 125; LDH, lactate dehydrogenase; FERR, ferritin; ALP, alkaline phosphatase; TBIL, Total Bilirubin; TP, Total Protein; ALB, albumin; α1-AT, α1-antitrypsin; Fib, fibrinogen; WBC, White Blood Cell; RBC, Red Blood Cell; HGB, hemoglobin; PLT, Platelet; BASO%, basophil percentage; hsCRP, high-sensitivity C-reactive protein; NLR, neutrophil-to-lymphocyte ratio; LMR, lymphocyte-to-monocyte ratio; PLR, platelet-to-lymphocyte ratio; FAR, fibrinogen-to-albumin ratio.

### 3.3 Nomogram-based predictive model for 1-year OS after surgery in HIV-CRC patients

**Figure 2:**
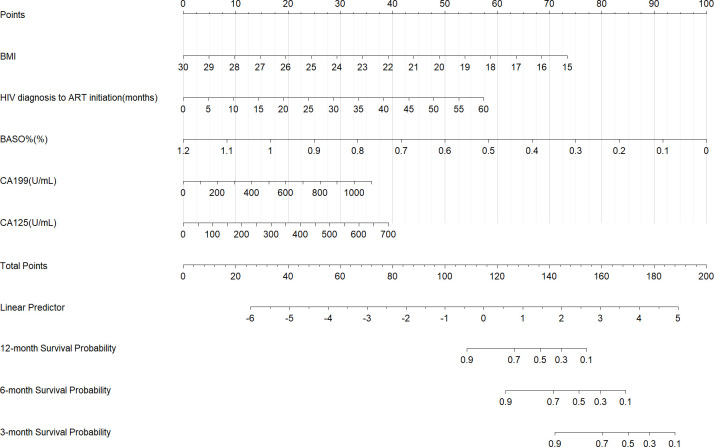
Nomogram-based predictive model for 1-year overall survival after surgery in HIV-CRC patients.

Using R 4.5.0 software, a preliminary nomogram model was constructed based on the five potential independent factors influencing 1-year OS postoperatively to estimate the 1-year OS rate for HIV-CRC patients (**Figure 2**). In the nomogram, each variable was assigned a value according to the patient’s actual condition, and a perpendicular line is drawn to the “Points” axis to yield the corresponding score. By summing the scores of all variables, the “Total Points” value is obtained. Subsequently, a vertical line is drawn downward from the total points to the “1 year survival probability” axis at the bottom, indicating the predicted 1-year OS rate for the specific HIV-CRC patients after surgery.

### 3.4 Evaluation of discrimination, calibration, and clinical validity in a nomogram prediction model

**Figure 3:**
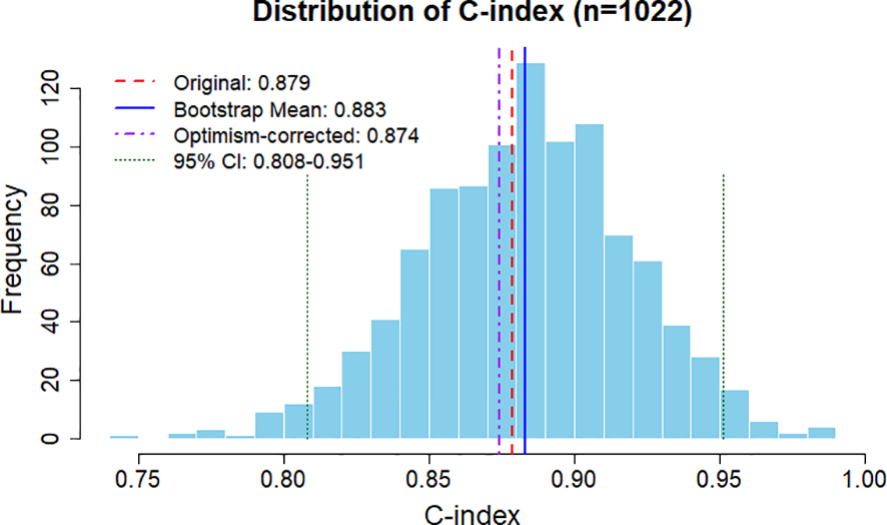
Bootstrap internal validation of the nomogram-based predictive model.

Bootstrap internal validation (n = 1022 resamples) was performed to assess the overall discriminative ability of the model across the entire follow-up period. The results showed a raw C-index of 0.879 and an optimism-corrected C-index of 0.874 (95%*CI* = 0.808 ~ 0.951), with an optimism of 0.005, suggesting favorable overall discrimination and minimal overfitting (**Figure 3**).

**Figure 4:**
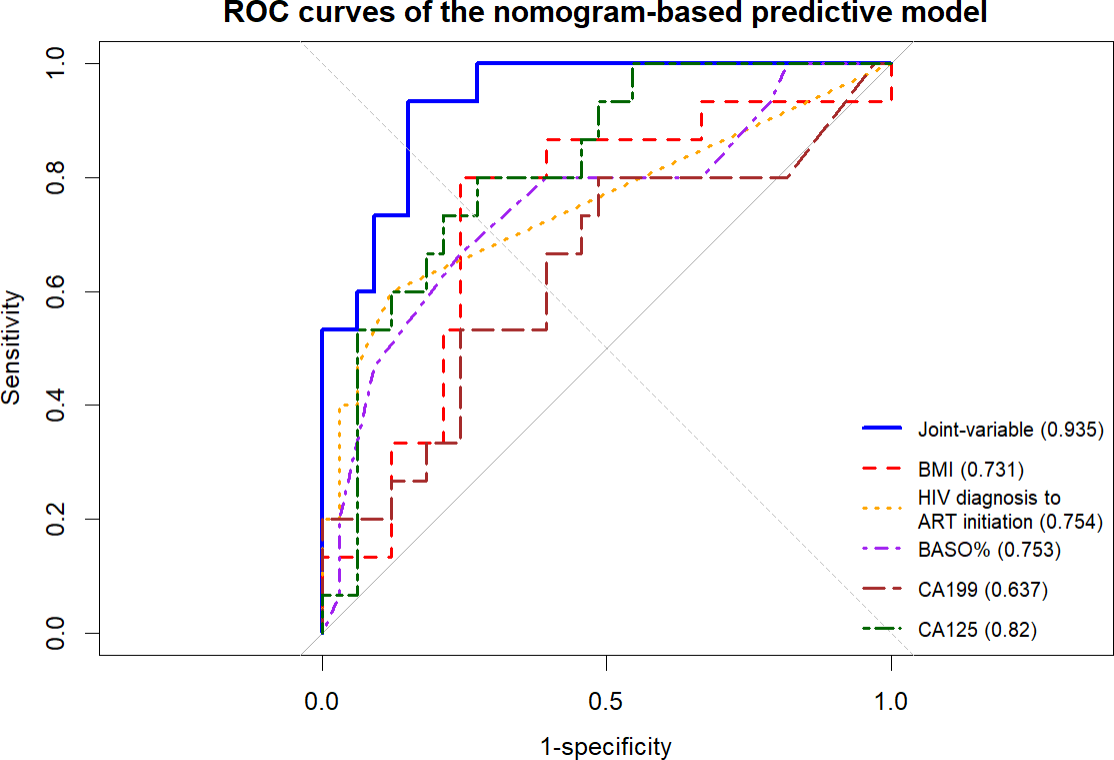
ROC curves of the nomogram-based predictive model. Comparison of predictive performance between single-variable and joint-variable models at 12 months.

ROC curves of the nomogram model were plotted for exploratory assessment of predictive performance at the 1-year time point. The area under the curve (AUC) values of each independent predictor were as follows: BMI (0.731), interval time from HIV diagnosis to ART initiation (0.754), BASO% (0.753), CA19-9 (0.637), and CA125 (0.820). The nomogram model incorporating the above five variables achieved an AUC of 0.935 (95%*CI* = 0.871 ~ 0.999), suggesting potentially improved discrimination compared with single-variable models, though this requires external validation (**Figure 4**).

**Figure 5:**
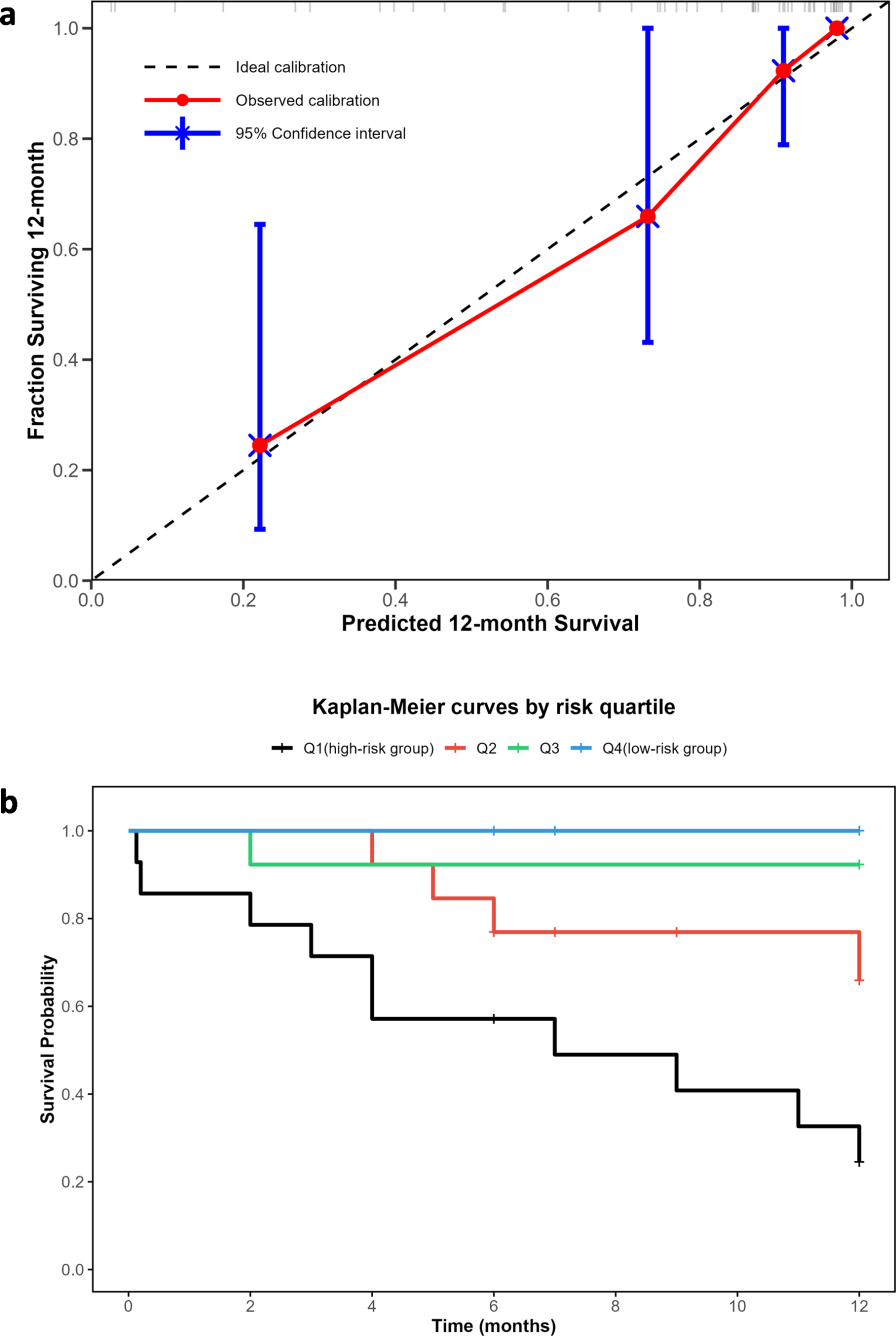
Calibration curves, and Kaplan−meier survival curves stratified by risk quartiles of the nomogram-based predictive model. **(a)** Calibration Curves; **(b)** Kaplan−Meier Survival Curves Stratified by Risk Quartiles.

Calibration curves were used to evaluate the calibration of the nomogram model. The predicted probabilities showed reasonable agreement with actual observed probabilities, with the calibration curve approaching the ideal 45° line (**Figure 5a**). Patients were stratified into four risk groups based on the nomogram risk scores. The 1-year survival rates were 24.5% (95%CI = 0.093 ~ 0.645) in Q1 (high-risk group, n = 14), 65.9% (95%CI = 0.431 ~ 1) in Q2 (n = 13), 92.3% (95%CI = 0.789 ~ 1) in Q3 (n = 13), and 100% (0 events) in Q4 (low-risk group, n = 14). Kaplan-Meier survival curves revealed significant differences in survival among the risk groups (Log-rank *χ²* = 25.7, *P* < 0.001), especially between Q1 and Q4 (Log-rank *χ²* = 15.9, *P* < 0.001) (**Figure 5b**).

**Figure 6:**
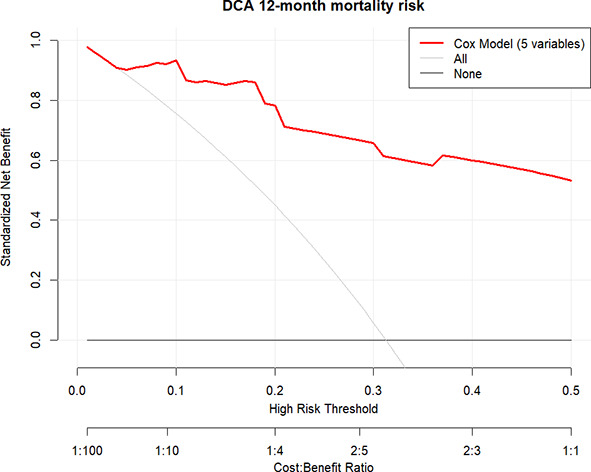
DCA curves of the nomogram-based predictive model.

The DCA curves of the nomogram prediction model demonstrated that the model provided positive net benefit across most threshold probabilities. Moreover, the proposed nomogram outperformed both “treat-all” and “treat-none” strategies, thus suggesting potential clinical utility, though prospective validation is needed to determine actual clinical value (**Figure 6**).

## 4 Discussion

Using COX regression analysis, this study identified BMI, interval time from HIV diagnosis to ART initiation, CA19-9, CA125, and BASO% as independent predictors of 1-year OS after surgery. Based on these factors, we developed a preliminary nomogram prediction model for exploratory assessment of postoperative prognosis in HIV-CRC patients.

This study identified the interval time from HIV diagnosis to ART initiation as a key prognostic factor specific to HIV-CRC patients, and a shorter interval was associated with prolonged survival. Although CD4^+^ T-cell count did not demonstrate statistical significance for prognostic differentiation in our cohort, the median CD4^+^ T-cell count was higher in the survivor group than in the non-survivor group. This suggests that earlier ART initiation may contribute to immune recovery and reconstruction, thereby potentially controlling tumor recurrence (18).

Several studies have reported that higher BMI is associated with better survival outcomes in CRC patients (19). This protective effect may be attributed to the fact that higher BMI reflects better nutritional reserve and metabolic buffering capacity, which help patients tolerate surgical stress and subsequent antitumor therapy (20). Furthermore, in the context of HIV infection, higher BMI may indicate better immune function and viral control. The elevated CA19−9 as an independent risk factor for postoperative prognosis in CRC patients was consistent with existing literature (21). As a cell−surface glycoprotein, CA19−9 participates in tumor cell adhesion and vascular invasion, and its high expression reflects higher tumor burden and stronger invasiveness. In HIV patients, elevated CA19−9 may suggest a synergistic effect between tumor progression and immunosuppressive status, leading to poorer prognosis. Although CA125 was traditionally regarded as a marker for ovarian cancer, recent studies have confirmed its important value in the prognostic evaluation of CRC (22). Studies have reported that patients with elevated preoperative CA125 have significantly shorter overall survival. Elevated CA125 may be associated with peritoneal metastasis, advanced tumor and lymph node stages, and poor differentiation, reflecting the peritoneal spreading tendency and invasive phenotype of the tumor.

Our exploratory analysis suggests that higher BASO% may be an independent protective factor for 1-year postoperative OS in HIV-CRC patients, though this novel finding requires confirmation in independent cohorts. Previous studies have shown that decreased preoperative basophil count is significantly associated with higher TNM stage, vascular and perineural invasion, and poor prognosis, affecting disease−free survival (23). Basophils regulate immune responses in the tumor microenvironment by secreting cytokines, and their infiltration has been related to an enhanced antitumor immune response. In HIV−infected individuals, higher basophil levels may indirectly reflect preserved host immune regulatory capacity, thereby helping to control tumor progression.

This study also analyzed the impact of three factors related to HIV infection on the prognosis of HIV-CRC patients, including preoperative antiviral regimens, the interval time from ART initiation to surgery, and the CD4^+^/CD8^+^ T-cell ratio. Although none of these factors reached statistical significance, baseline characteristics revealed that patients in the survivor group were more like to have received preoperative ART, exhibited a longer median interval from ART initiation to surgery, and had a higher median CD4^+^/CD8^+^ T-cell ratio. Consistent with the observed impact of delayed ART initiation on survival, these results suggest that initiating and maintaining regular ART treatment promptly after HIV diagnosis, thereby leading to immune restoration as reflected by a higher CD4^+^/CD8^+^ T-cell ratio, may contribute to improved clinical outcomes in HIV-CRC patients. However, due to the lack of routine HIV RNA monitoring in most cases, the impact of HIV viral replication on CRC prognosis could not be assessed.

Currently, there is limited research on the prognosis of HIV-CRC patients. In recent years, machine learning−based postoperative prediction studies have provided important insights for constructing prognostic models using routine clinical and laboratory variables. As demonstrated in the study on patients undergoing intestinal obstruction surgery, machine learning models constructed using routinely available clinical indicators can effectively predict major postoperative complications to a certain extent (24). This study proved the value of routine clinical data in postoperative risk prediction, providing external support for our selection of readily available predictive variables including BMI, CA19−9, CA125, BASO%, and interval time from HIV diagnosis to ART initiation. In this context, this study employed multiple statistical methods for the exploratory analysis of independent prognostic factors and preliminarily constructed a nomogram for 1-year OS prediction. The model showed favorable discrimination and calibration in our single−center retrospective cohort, suggesting that it could serve as a potential tool for early prognostic evaluation in HIV−CRC patients. Nevertheless, as a single−center retrospective study, our findings were limited by the relatively small sample size and short follow−up duration (25, 26). These results should be considered preliminary and exploratory, and rigorous external validation is mandatory before routine clinical implementation (27). Future external validation in multi−center cohorts with extended longitudinal data is warranted to evaluate the generalizability and robustness of the model across different patient populations.

## 5 Conclusion

Based on these findings, we developed a preliminary nomogram prediction model incorporating five independent prognostic factors for exploratory prediction of 1-year postoperative OS: BMI, interval time from HIV diagnosis to ART initiation, CA19−9, CA125, and BASO%. This model was subsequently validated using ROC curves, calibration curves, and decision curve analysis, all of which confirmed its potential utility in estimating 1-year postoperative survival probability in HIV-CRC patients. The potential value of this nomogram lies in providing a reference for individualized management of HIV−CRC patients, helping to identify high−risk patients at an early stage and thus guiding individualized adjustments to the intensity of perioperative care and the frequency of postoperative monitoring. Notably, because it relies on easily accessible biomarkers such as BMI, CA19−9, CA125, and BASO%, it can be used as a potential risk stratification tool even in resource−limited settings.

## Data Availability

The original data of this study involve patient privacy and have not been publicly included in the article/supplementary material. For further inquiries, please contact the corresponding author.
